# Cognitive Control, Not Time, Determines the Status of Items in Working Memory

**DOI:** 10.5334/joc.98

**Published:** 2020-04-09

**Authors:** Jacqueline M. Fulvio, Bradley R. Postle

**Affiliations:** 1 Department of Psychology, University of Wisconsin–Madison, Madison, Wisconsin, US; 2 Department of Psychiatry, University of Wisconsin–Madison, Madison, Wisconsin, US

**Keywords:** working memory, cognitive control, TMS

## Abstract

Despite the fact that multiple items can be held in working memory (WM), it is often the case that only one of these is relevant for guiding in-the-moment behavior. Therefore, understanding how priority is established and controlled in WM is an important problem. Data from Rose et al. (2016) have provided evidence that although neuroimaging evidence for an active trace of an “unprioritized memory item” (UMI) held in WM drops to baseline levels, evidence for its retention in WM can be “reactivated” by a single pulse of transcranial magnetic stimulation (TMS). Critically, this TMS-reactivation effect was specific to the first delay period of a dual serial retrocue (DSR) task, when the UMI could be needed for the trial’s second memory probe, and was not observed during the second delay period, when the uncued item was no longer needed (i.e., when it is an “irrelevant memory item” [IMI]). A problem for the interpretation of these results, however, is that the status of the UMI/IMI was confounded with time spent in WM, as well as with the number of intervening cognitive operations. Here, we report data from a follow-up study designed to replicate the findings Rose et al. (2016) and to add a condition that unconfounds time-since-sample-presentation and UMI/IMI status. The results indicate that the TMS-reactivation effect is, indeed, an index of status in WM (UMI vs. IMI), and not a mere consequence of time elapsed since sample presentation.

## Introduction

An important question in working memory (WM) research is how we maintain multiple items in WM and dynamically prioritize them according to task demands (Myers et al., 2017). One line of cognitive neuroscience research has addressed this with a dual serial retrocuing (DSR) task in which two sample items are followed, after an initial delay, by a retrocue indicating which of the two will be the first to be tested. After that test (either recall or recognition) a second retrocue indicates, with equal probability, which of the samples will be tested by a second test. This design creates a situation in which, during the delay between the first retrocue and the first memory test, one item has the status of “prioritized memory item” (PMI; which we assume occupies the focus of attention) and the other the status of “unprioritized memory item” (UMI; still in WM, but possibly in a different attentional state). The core finding, from functional magnetic resonance imaging (fMRI) and electroencephalography (EEG) studies, has been that multivariate evidence for an active representation of a sample item drops to baseline when it takes on the status of UMI (Lewis-Peacock et al., 2012; LaRocque et al., 2013; LaRocque et al., 2017). This suggests that the UMI may transition to a representational state that is different from when it is attended. (Note that more recently there have been reports of evidence for an active neural representation of the UMI [e.g., van Loon et al., 2018; Yu & Postle, 2018; Christophel et al., 2018; Rademaker et al., 2019; Wan et al., preregistered], but this development in the literature is not directly relevant for the present study.)

A series of experiments by Rose et al. (2016) was designed to address two possible accounts of the change of representational state of the UMI. One account is that the UMI is transferred to long-term memory (LTM), then later retrieved if cued by the second retrocue. A second is that encoding an item into WM entails the simultaneous creation of two traces – an activity-based trace and a synaptic weight-based trace (c.f., Masse et al., 2019) – and that the transition to UMI entails the dissipation of the former but not the latter. Rose et al. (2016) recorded the EEG while subjects performed a DSR task, and delivered single pulses of transcranial magnetic stimulation (spTMS) to regions in posterior cortex, predicting that a synaptic trace of the UMI might be decodable from the spTMS-evoked response in the EEG, but that a LTM-based representation of the UMI should not be. The reasoning was that maintaining the UMI in WM necessarily requires retention of the binding between that item’s identity and the trial-specific context in which it had been presented, and if this binding is instantiated in elevated connectivity between neural representations of content and context, this might be revealed in the EEG by the filtering of the spTMS-evoked response. If, in contrast, the UMI is transferred to LTM, the consequent medial temporal lobe-based representation would not be expected to interact in a trial-specific way with the spTMS-evoked response.

Rose et al. (2016) were, indeed, successful in decoding the category of the UMI from the spTMS-evoked response with multivariate pattern analysis (MVPA). Importantly, this was only observed when spTMS was delivered prior to the first recognition probe, when the UMI was still potentially relevant for later in the trial, but not when spTMS was delivered during the delay period following the second retrocue, when subjects knew that the uncued item was no longer relevant for that trial. (Because the distinction between the uncued item following the first retrocue and the uncued item following the second retrocue is of central importance for the present study, we will distinguish between them by referring to the former as the UMI and the latter as the “irrelevant memory item” [IMI].) The interpretation was that the IMI had been removed from WM after the second retrocue, an operation that would include the removal of that item’s synaptic trace. (For a more detailed consideration of how information might be removed from WM, see Lewis-Peacock et al., (2018).) Finally, to assess whether there were functional consequences of UMI decodability from the spTMS-evoked response, Rose et al. (2016) carried out an additional experiment in which 40% of nonmatching recognition probes were the UMI (i.e., lures). Results of this final experiment indicated that spTMS delivered when the uncued item was a UMI was associated with an increased false alarm rate (FAR) to lures, relative to non-lure nonmatching probes, but that the same was not true when spTMS was delivered when the uncued item was an IMI.

Overall, the results from the Rose et al. (2016) were interpreted as consistent with the idea that the UMI remains in WM (and, therefore, under cognitive control), and inconsistent with the possibility that the UMI is transferred to LTM (c.f., LaRocque et al. 2015). A complication with this interpretation, however, is that, in all of the experiments in Rose et al., (2016), the status of the uncued item (UMI, IMI) was confounded with time spent in WM, as well as with the number of intervening cognitive operations: the UMI acquired its status with the onset of the first retrocue, whereas the IMI acquired its status several seconds further along in the trial, and after the first recognition probe, with the onset of the second retrocue. Thus, it remains possible that the selective effect of spTMS delivered when the item was a UMI does not provide evidence for the retention of the UMI in WM, but, rather, is merely a result of the recency with which it was presented, and/or the absence of intervening cognitive operations.

This Data Report presents the results of a follow-up study designed to replicate key elements from Rose et al. (2016), and to add a condition that did not confound UMI/IMI status with the theoretically uninteresting factors that complicate the interpretation of the results from Rose et al. (2016). We investigated the impact of delay-period spTMS on behavioral performance implemented in two working memory tasks, a DSR task like that used in Rose et al. (2016) and a single retrocue task in which subjects are cued and tested on the memory set only once. Across the two tasks, there were three conditions of interest concerning the impact of spTMS during (1) the delay period of the DSR task when the uncued item had the status of UMI; (2) the delay period of the DSR task when the uncued item had the status of IMI; and (3) the delay period of the single retrocue task, when the uncued item had the status of IMI. We emphasize the critical condition of (3), in which the uncued item has the same priority status as the IMI of the DSR task, but the same time-lag relative to the sample period as the UMI of the DSR task, thereby allowing us to unconfound cognitive-control versus time-lag accounts of the UMI-reactivation effect.

## Methods

### Subjects

Fourteen neurologically healthy members of the University of Wisconsin–Madison community, with no reported contraindications for magnetic resonance imaging (MRI) or transcranial magnetic stimulus (TMS), underwent a structural MRI scan and participated in three sessions of WM task performance with concurrent EEG and spTMS, all on separate days. Data from two individuals were excluded because of noncompliance with task instructions during the behavioral sessions, resulting in a final sample size of 12 (5 females, 18–28 years, *M* = 21.7 years, all right handed). An a priori power analysis based on the behavioral results of Experiment 4 of Rose et al. (2016) indicated that 12 subjects would be needed to achieve 80% power in the critical behavioral comparison of interest (described in more detail below). All subjects had normal or corrected-to-normal vision with contact lenses (eyeglasses were not compatible with TMS targeting apparatus), and all reported having normal color vision. The research complied with the guidelines of the University of Wisconsin–Madison’s Health Sciences Institutional Review Board. All subjects gave written informed consent at the start of each session and received monetary compensation in exchange for participation.

### Experimental procedure

**Figure 1:**
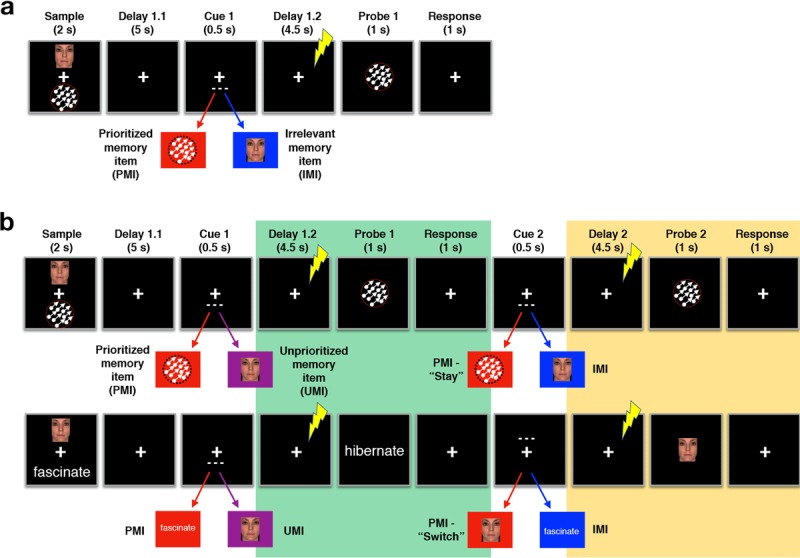
**Experimental details. (a)** Single retrocue task. Two items, each from one of three categories (faces, translating dots, words) appeared simultaneously for two seconds above and below fixation. After a 5-second delay (Delay 1.1), a dashed line (Cue 1) appeared above or below fixation to indicate which of the two items would be probed for response. The cue was followed by a 4.5-second delay (Delay 1.2), during which a single pulse of TMS was delivered, 2–3 seconds after the offset of the cue, on 50% of trials. After Delay 1.2 a probe stimulus appeared at fixation and subjects indicated whether the probe stimulus was a match or nonmatch to the probed memory item. **(b)** Dual serial retrocue (DSR) task. The task structure was identical to the single retrocue task until the (first) response period, after which a second cue appeared that referred to the PMI on 50% of trials (upper example) or the UMI on 50% of trials (lower example). spTMS was delivered during Delay 2 on 50% of trials.

The experiment was carried out in four sessions, each on a separate day. The first session was the MRI scan, which yielded the anatomical image used to guide spTMS during the three behavioral sessions. During each behavioral session subjects performed two WM tasks, a DSR task and a single retrocue task (see Figure 1). Tasks were blocked, with each session comprising eight 30-trial blocks, alternating between DSR and single retrocue. The order of the blocks was switched across session within subjects, and counterbalanced across subjects. (The number of sessions was chosen to acquire the desired number of trials per cell in the experimental design with sessions whose length would be tolerated by subjects).

### Behavioral tasks

The single retrocue task (Figure 1a) began with the simultaneous presentation of two sample items drawn from two of three categories (face, word, direction of dot motion), one above and one below central fixation (2 sec), followed by 5 sec of fixation (“Delay 1.1”), followed by a cue (dotted line) whose location indicated which of the two samples would be tested, followed by 4.5 sec of fixation (“Delay 1.2”), followed by a recognition probe (1 sec). Responses were to be made within a 2-sec window beginning with probe onset, and ITI varied between 2–4 sec. Upon registration of the response, feedback was provided (the fixation cross turned green for correct responses, red for incorrect responses) for the remainder of the response window. 50% of probes were matches of the cued item, 30% were nonmatches drawn from the same category as the cued item, and 20% were the uncued item from that trial’s memory set (i.e., lures). Subjects were not explicitly told that the uncued memory item could appear as lures. Throughout each block the TMS coil was positioned to target area IPS2 in the right hemisphere, and randomization of memory set, cued category, and probe type were constrained so that spTMS was delivered on 50% of trials of each type.

The DSR task (Figure 1b) replicated the procedure of the single retrocue task, but with a second cue (“Cue 2”) following “Probe 1” with an SOA of 2.5 sec, followed by “Delay 2” (4.5 sec) and “Probe 2” (1 sec, plus additional 1 sec response capture window). Cue 2 appeared in the same location as Cue 1 on 50% of trials, and spTMS was counterbalanced across all levels of Cue 1, Probe 1, Cue 2 and Probe 2.

### Stimuli

The experimental stimuli were the same as those used in Rose et al. (2016) and presented in MATLAB using the Psychophysics Toolbox (Brainard, 1997; Pelli, 1997; Kleiner et al., 2007) on an LCD with a resolution of 1920 × 1080 and background color set to black. The stimuli were viewed from a 70 cm viewing distance. Face stimuli were selected from the set used by Rose et al. (2016; see also Rose et al., 2012). The images comprised non-famous faces with a neutral expression selected from publicly available photographs and constructed using FaceGenModeller software (https://facegen.com/modeller.htm). The face images were standardized in terms of head position and size, and the images were cropped to exclude hair and other salient features such as facial hair and glasses. At the start of each experimental block, a subset of the set of faces was chosen to be the stimuli for the block’s face-containing trials, thus pre-defining the sample and nonmatching face for each one. Word stimuli were also selected from the set used by Rose et al. (2016). The word list was created by first generating a list of three-syllable sample words using the English Lexicon Project Word List Generator (https://elexicon.wustl.edu/; Balota et al., 2007). A second list of matched rhyming three-syllable words was then generated using the word generator at rhymer.com, and finally a third list was generated containing lexically-similar but nonmatching rhyming words. As with the face stimuli, a subset of the word list was chosen at the start of each experimental block, thus pre-defining the sample, matching, and nonmatching word for each word-containing trial. Dot motion stimuli contained ~125 white dots, each subtending ~0.14 degrees of visual angle (DVA), and were presented in a circular aperture ~8.3 DVA in diameter. The dots moved with 100% coherence at a speed of 3 deg/s for the duration of the stimulus presentation (2 sec) in a random direction chosen on a trial-by-trial basis from the full 360 degree range. For face and motion categories, subjects judged whether probes were precise matches. For nonmatch probes that were not lures, face probes were created by morphing the sample face in the FaceGenModeller software with one of the other faces of the same gender so that the probe face comprised a 50–70% morph, and the direction of dot-motion probes differed from the sample by a range of 5–45 degrees (clockwise or counterclockwise, randomly determined). For the word category, the probes were always different from the sample, and subjects judged whether or not the probe rhymed with the sample. Nonrhyming word probes were chosen from the pre-defined nonmatching word list selected at the start of the experimental block.

### MRI data acquisition and preprocessing

Whole brain images were acquired with a 3T MRI scanner (Discovery MR750; GE Healthcare) at the Lane Neuroimaging Laboratory at the University of Wisconsin–Madison. High resolution T1-weighted images were acquired for all subjects with an FSPGR sequence (8.2 ms repetition time (TR), 3.2 ms echo time (TE), 12° flip angle, 172 axial slices, 256 × 256 in-plane, 1.0 mm isotropic). The T1-weighted images were processed using the AFNI software program to align each subject’s brain with the MNI152_T1_1mm template. In AFNI, a mark was inserted in right intraparietal sulcus (rIPS2; coordinate: –22 70 58) and used as the target for spTMS (see below).

### spTMS targeting and stimulation

**Figure 2:**
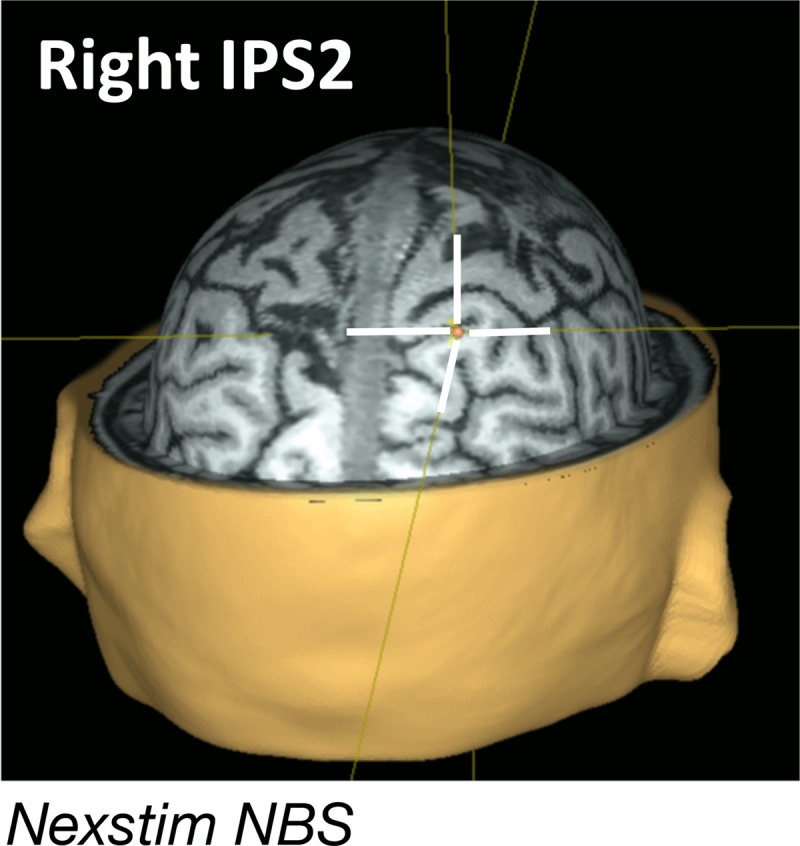
**Single-pulse TMS administration.** spTMS was administered to rIPS2 (coordinate: –22 70 58) localized for each subject using an anatomical scan and NexStim Navigated Brain Stimulation software. spTMS was delivered unpredictably on 50% of trials during Delay 1.2 of the DSR task, on 50% of trials during Delay 2 of the DSR task, and on 50% of trials during Delay 1.2 of the single retrocue task.

IPS2 was selected as a target for spTMS because of its role in implementing priority maps (e.g., Jerde et al., 2012; see Figure 2), and in binding content to context in visual working memory (Gosseries, Yu, et al., 2018). The reasoning was as follows: if the UMI is held in WM, this would involve a network including IPS2 to maintain elevated connectivity between the neural representations of sample identity and the location at which the sample had been presented. Furthermore, removing an item from working memory, as we hypothesize happens to the IMI, would entail the return of this state of IPS2-to-stimulus connectivity to baseline levels. Therefore, reactivation of the UMI by delay-period spTMS to IPS2 would provide evidence for its retention in WM. The alternative account – that the UMI is transferred to LTM – would not predict reactivation by spTMS to IPS2 because retaining recently encoded information in LTM does not entail sustained involvement of the parietal priority map.

spTMS targeting was achieved with a navigated brain stimulation (NBS) system that uses infrared-based frameless stereotaxy to coregister the location and position of the subject’s head and that of the TMS coil according to the individual’s high-resolution MRI (NexStim eXimia, Helsinki, Finland). spTMS was delivered with an eXimia TMS Focal BiPulse transcranial magnetic stimulator fit with a figure-of-eight stimulating coil. NBS allows estimation of the electrical field induced by TMS at the cortical target using a model of the subject’s head, information about the coil position, and the distance from the coil to the cortical target. spTMS was delivered to the target to achieve an estimated intensity at the stimulation target of 90–110 V/m (60–75% of stimulator output, depending on the thickness of the subject’s scalp, cortex and depth of the target). The coil was oriented along the sagittal plane to induce an anterior-posterior direction of current, with individual adjustments to minimize EEG artifact. Stimulator intensity, coil position, and coil orientation were held constant for each subject for the duration of each session. To mask the sound of TMS coil discharge, subjects were fitted with earbuds through which white noise was played during task blocks, with volume titrated such that the subjects could not detect the click produced by coil discharge. Stimulation parameters were in accordance with published safety guidelines.

### Data analysis

Behavioral effects of spTMS were analyzed with Bayes factor hypothesis testing (e.g., Berger, 2006; Jeffreys, 1935, 1961; Kass & Raftery, 1995; Wagenmakers et al., 2017) using the JASP open-source software package (https://jasp-stats.org/; JASP Team, 2016). First, we planned to assess evidence for a replication of the critical finding from Rose et al. (2016), an elevated false-alarm rate (FAR) to lures for spTMS delivered during Delay 1.2, but not during Delay 2, of the DSR task. Next, the principal question of interest for this experiment was whether Delay 1.2 spTMS would also produce an elevated FAR to lures on the single retrocue task. Comparison across tasks would be implemented by examining evidence for a Delay-1.2-spTMS (delivered/not delivered) × task (DSR/single retrocue) interaction.

## Results

**Figure 3:**
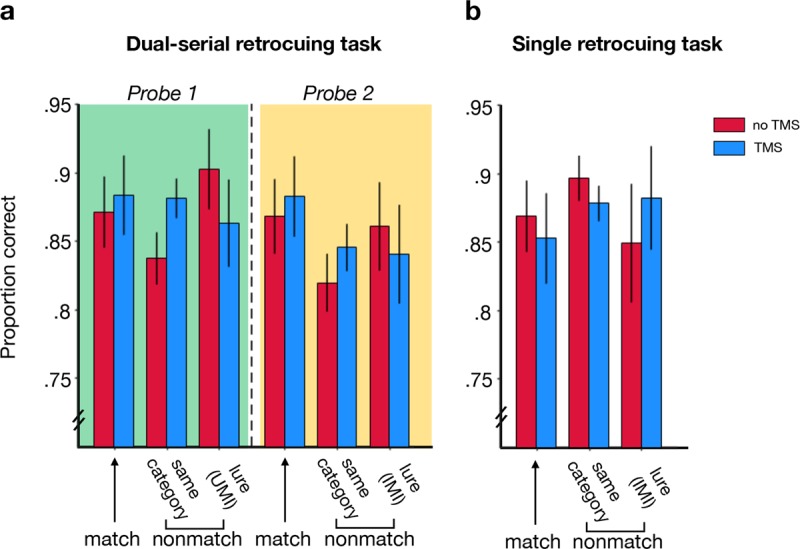
**Behavioral performance in the experimental tasks. (a)** Accuracy on the DSR task as a function of probe type (match, nonmatching item drawn from same category as the PMI, and nonmatching items that are lures) and spTMS condition. **(b)** Accuracy on the single retrocue task as a function of probe type and spTMS condition. Error bars correspond to +/– 1 standard error of the mean.

Behavioral performance on the DSR task qualitatively replicated the pattern from Rose et al. (2016; see Figure 3a), with moderate evidence that spTMS during Delay 1.2 elevated the false-alarm rate to UMI lures at Probe 1 (relative to no spTMS; *BF_–0_* = 3.39), and only a weak trend in this direction for IMI lures following spTMS during Delay 2 (*BF_–0_* = 0.998). Critically, spTMS during Delay 1.2 of the single retrocue task did not produce an increased false alarm rate to the uncued item (*BF_–0_* = 0.12; Figure 3b), and the interaction of Delay 1.2 spTMS (delivered/not delivered) × task (DSR/single retrocue) provided moderate evidence for a difference in the status of the uncued item during Delay 1.2 in the two tasks (*BF_10_* = 9.26).

## Discussion

We investigated the impact of delay-period spTMS on behavioral performance in three conditions implemented in two working memory tasks: (1) during Delay 1.2 of the DSR task when the uncued item had the status of UMI; (2) during Delay 2 of the DSR task when the uncued item had the status of IMI; and (3) during Delay 1.2 of the single retrocue task, when the uncued item had the status of IMI. Results indicated that only in condition (1), when the uncued item had the status of UMI, did spTMS produce an elevated FAR to lures. Critically, conditions (1) and (3) were matched for time elapsed between sample offset and spTMS, meaning that status in the trial – a UMI that might be needed later in the trial vs. an IMI that was no longer relevant for that trial – was the critical factor determining the effect of spTMS. We interpret these results as consistent with two propositions about the prioritization of information in WM. First, information that has the status of UMI is held in WM via elevated connectivity between neural representations of the item and the context in which it was presented. Second, the control of the contents of WM includes the ability to remove an item from WM the moment it is no longer relevant for behavior (c.f., Postle and Oberauer, in press). At the present time, this second proposition only applies to the removal of content while a task is underway because a recent study has reported that spTMS of the prefrontal cortex (PFC) during the intertrial interval (ITI) of a visual working memory task enhances the serial bias effect (Barbosa et al., in press). That is, because ITI spTMS of PFC increased the influence of the content from the preceding trial on the current one, it may have had the effect of reactivating the presumably no-longer-relevant information from the previous trial, an explanation that would be consistent with complementary findings from neural network modeling and from extracellular recordings from the PFC of nonhuman primates (Barbosa et al., in press). Because the results from the present study provide evidence against the reactivation of the IMI during the single retrocue task, their juxtaposition with the findings of Barbosa et al. (in press) raise the tantalizing possibility that the processing of no-longer-relevant stimulus information may differ as a function of whether its status changed during the trial or as the simple consequence of the trial being completed.

## Data Accessibility Statement

Data and related information available at: https://osf.io/qrh4e/.
